# Spontaneous hemothorax caused by rivaroxaban treatment for pulmonary embolism: A case report

**DOI:** 10.1002/ccr3.8333

**Published:** 2023-12-11

**Authors:** Bassem Al Hariri, Ahmad Taher Alharafsheh, Muad Abdi Hassan, Abdulqadir J. Nashwan, Mohamed Gaafar Mohamedali, Hatem Mabrouk Abusriwil

**Affiliations:** ^1^ Department of Medicine Hamad Medical Corporation Doha Qatar; ^2^ College of Medicine Qatar University Doha Qatar; ^3^ Department of Pharmacy Hamad Medical Corporation Doha Qatar; ^4^ Medical Education Department Hamad Medical Corporation Doha Qatar; ^5^ Nursing Department Hamad Medical Corporation Doha Qatar

**Keywords:** acute medicine, anticoagulation, direct oral anticoagulants (DOACs), general medicine, hematology, pulmonary embolism, radiology & imaging, respiratory medicine, rivaroxaban, spontaneous hemothorax, vitamin K antagonists (VKAs)

## Abstract

Hemothorax is a rare and potentially fatal condition characterized by pleural effusion containing over 50% of the patient's hematocrit. A massive hemothorax involves blood loss exceeding 1.5 L. Common causes include chest trauma, invasive thoracic procedures, anticoagulant medications, vascular anomalies, malignancies, and hematologic abnormalities. Spontaneous hemothorax may be seen in conjunction with pulmonary infarction and spontaneous pneumothorax. Anticoagulation is a key therapeutic strategy for certain thromboembolic events, such as pulmonary embolism. Historically, these events were treated with vitamin K antagonists (VKAs), which have demonstrated variable plasma concentrations and an increased risk of hemorrhage. With the advent of direct oral anticoagulants (DOACs), treatment has become as effective as VKAs while significantly reducing the risk of hemorrhage. However, some researchers have speculated that hemorrhagic complications in certain cases could be worse with DOACs than with VKAs. In the case presented here, we identified a genuine association between the use of rivaroxaban and spontaneous hemothorax following the initiation of treatment for pulmonary embolism.

## Graphical abstract

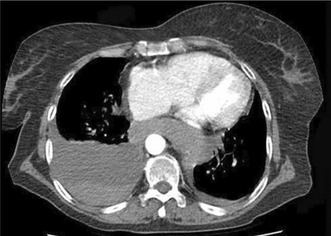

## 1 INTRODUCTION

Hemothorax, a rare and potentially fatal condition, occurs when pleural effusion has a hematocrit exceeding 50% of the patient's blood. Massive hemothorax involves blood loss greater than 1.5 L.^1^ Common causes include chest trauma, invasive thoracic procedures, anticoagulant medications, vascular anomalies, malignancies, and hematologic abnormalities.^2^, ^3^ Spontaneous hemothorax can be associated with pulmonary infarction and spontaneous pneumothorax.^4^

Anticoagulation therapy is integral to managing certain thromboembolic events, such as pulmonary embolism. These events were previously treated using vitamin K antagonists (VKAs), which display unpredictable plasma concentration fluctuations and an increased risk of bleeding.^5^ However, introducing direct oral anticoagulants (DOACs) offers a treatment option as effective as VKAs while significantly reducing the risk of hemorrhage.^5^ Despite rivaroxaban's very modest risk of bleeding, the recent cases in the literature demonstrate that spontaneous hemothorax may develop after using NOACs while they have a low bleeding risk for it.^2^

In this case, we identified a clear association between rivaroxaban use and the onset of spontaneous hemothorax following the initiation of treatment for pulmonary embolism.

## 2 CASE PRESENTATION

A 49‐year‐old man from Bangladesh presented with complaints of chest pain on the right side, shortness of breath, and cough. These were similar to his previous hospitalization when he was diagnosed with acute pulmonary embolism (PE), deep vein thrombosis (DVT) in his left leg, and May–Thurner syndrome in his left common iliac vein. The patient confirmed that he had been taking rivaroxaban as prescribed.

The patient reported having right‐sided pleuritic chest pain, breathlessness, and an occasional cough since he was discharged. He started experiencing these symptoms 1 day before he was admitted as a case of hemorrhagic pleural effusion, which is still being investigated. This information was provided in the referral letter from primary care.

**FIGURE 1:**
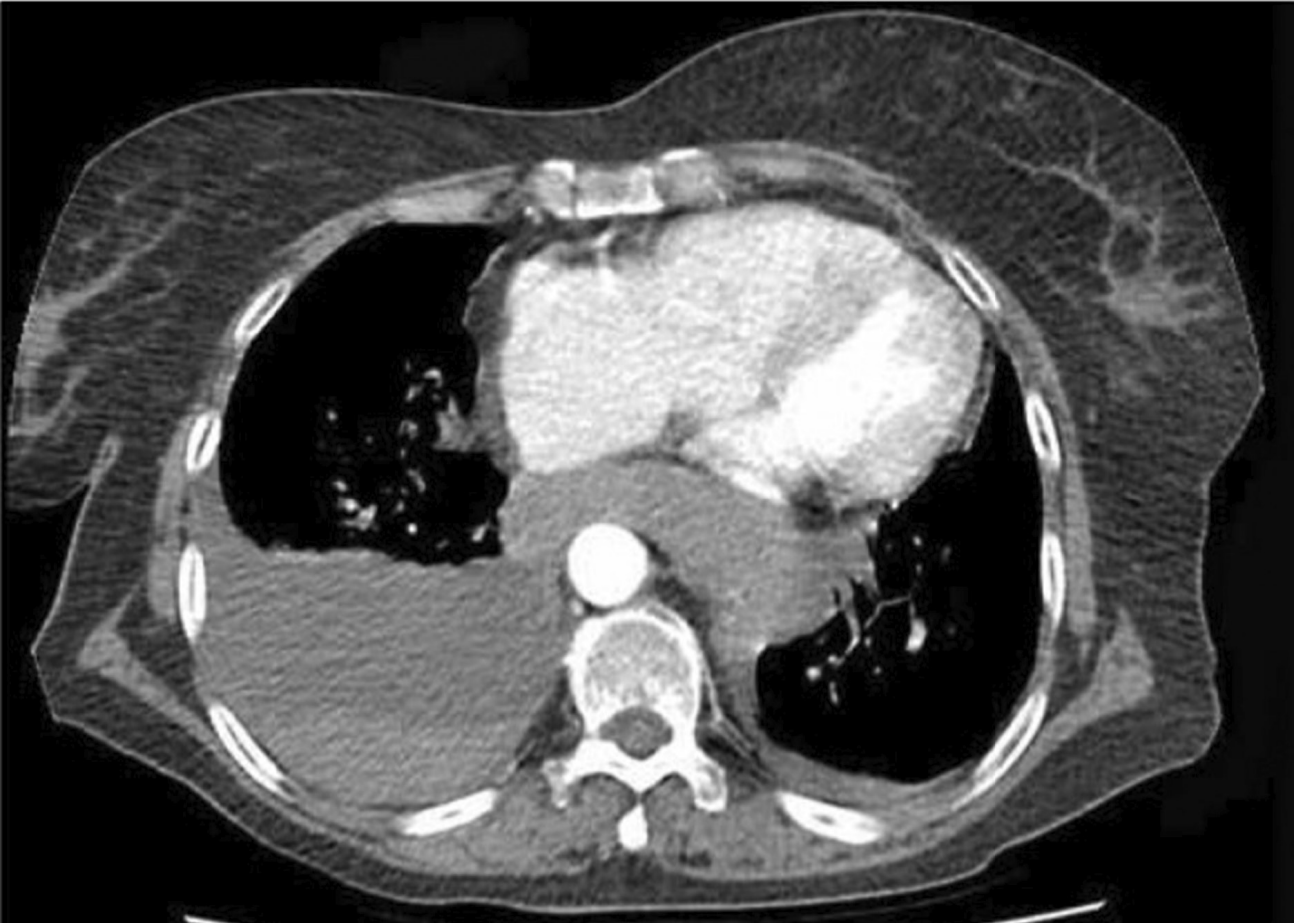
Chest CT reveals right‐side pleural effusion and mediastinal hematoma.

The differential diagnosis on admission was PE recurrence; pleural effusion is not related to PE, so the team continued rivaroxaban and CT pulmonary angiogram (CTPA) showed left lung lower lobe segmental pulmonary arterial thromboembolism and moderate right‐side pleural effusion associated with atelectatic changes (Figure 1). Further evaluation showed moderate pleural effusion on the right side. Then the repeated CTPA did not show any new changes suggestive of recurrent PE. The pleural effusion was minimal to mild severity compared to the previous X‐ray.

**FIGURE 2:**
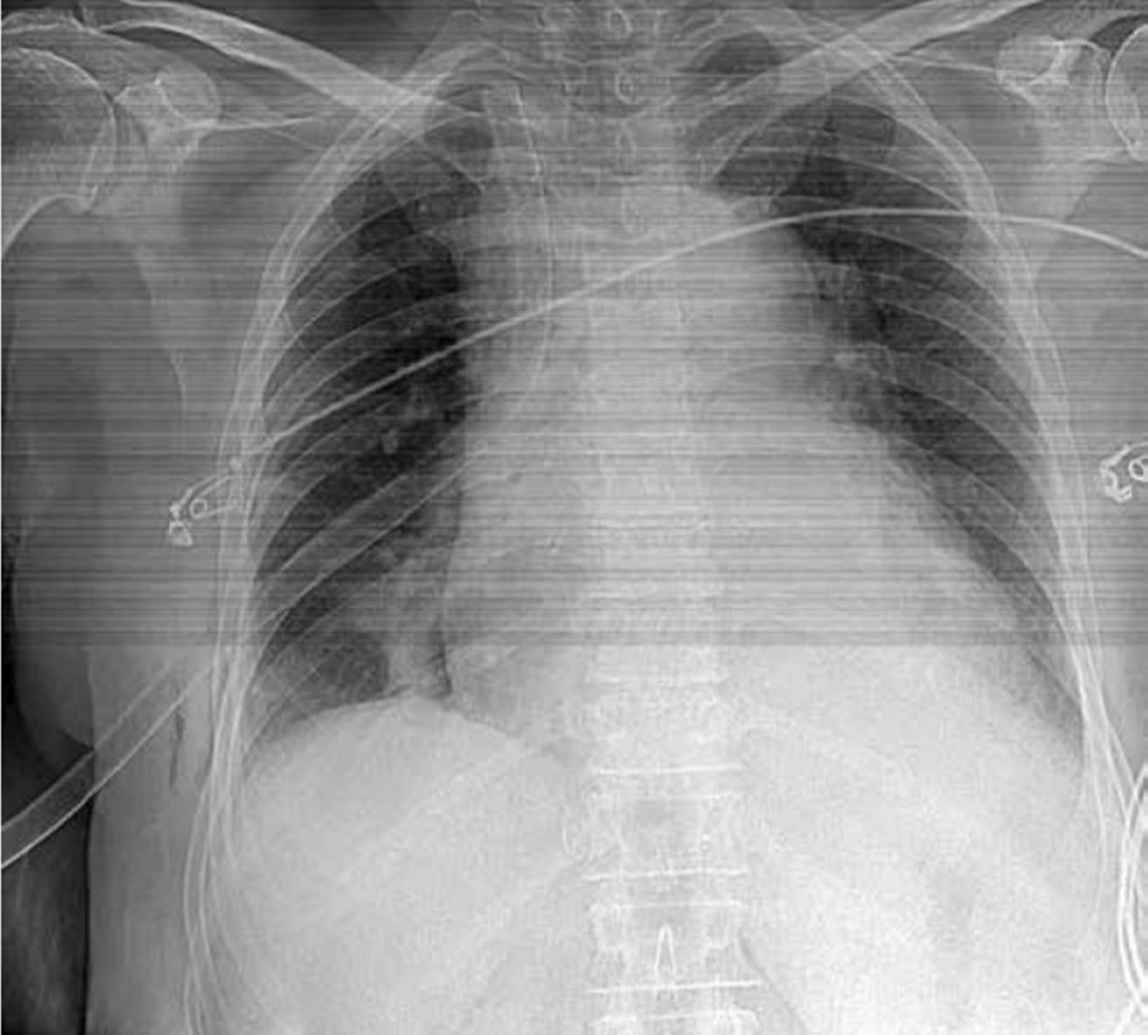
Chest X‐ray after tube thoracostomy.

On the third day of admission, diagnostic thoracentesis was planned. Rivaroxaban was stopped, INR was 1.7, and Hb was 12.6. Diagnostic pleural aspiration under the US showed heavy hemorrhagic pleural effusion (see Table 1), and CXR post‐procedure showed a further increase in the amount of effusion. After that, the patient went for chest tube insertion and switched his medication regime to heparin infusion for 24 h and monitored his Hb levels or if any complications happened (Figure 2).

**TABLE 1:** Laboratory tests and pleural fluid analysis.

Test	Admission	Discharge	Normal reference ranges
WBC	12.1	11.2	4–10
RBC	4.9	4.5	4.5–5.5
HGB	14.0	12.6	13–17
HCT	41.1	37.3	40–50
MCV	83.9	83.6	83–101
MCH	28.6	28.3	27–32
MCHC	34.1	33.8	31–34
PLT	280	360	150–410
NEUTROPHIL	72.5	70.4	40–69
LYMPHOCYTE	16.0	19.4	30–40
ESINOPHIL	3.4	1.4	<1
PROTHROMPIN T	19.4	12.9	9.4–12.5
INR	1.7	1.2	1
APTT	26.8	25.8	25.1–36.5
PLEURA fluid analysis			
TNC	2250		
RBC	3177500		
NEUTROPHIL PF	20.0		
LYMPHOCYTE PF	53.0		
HCT PF	29		

**FIGURE 3:**
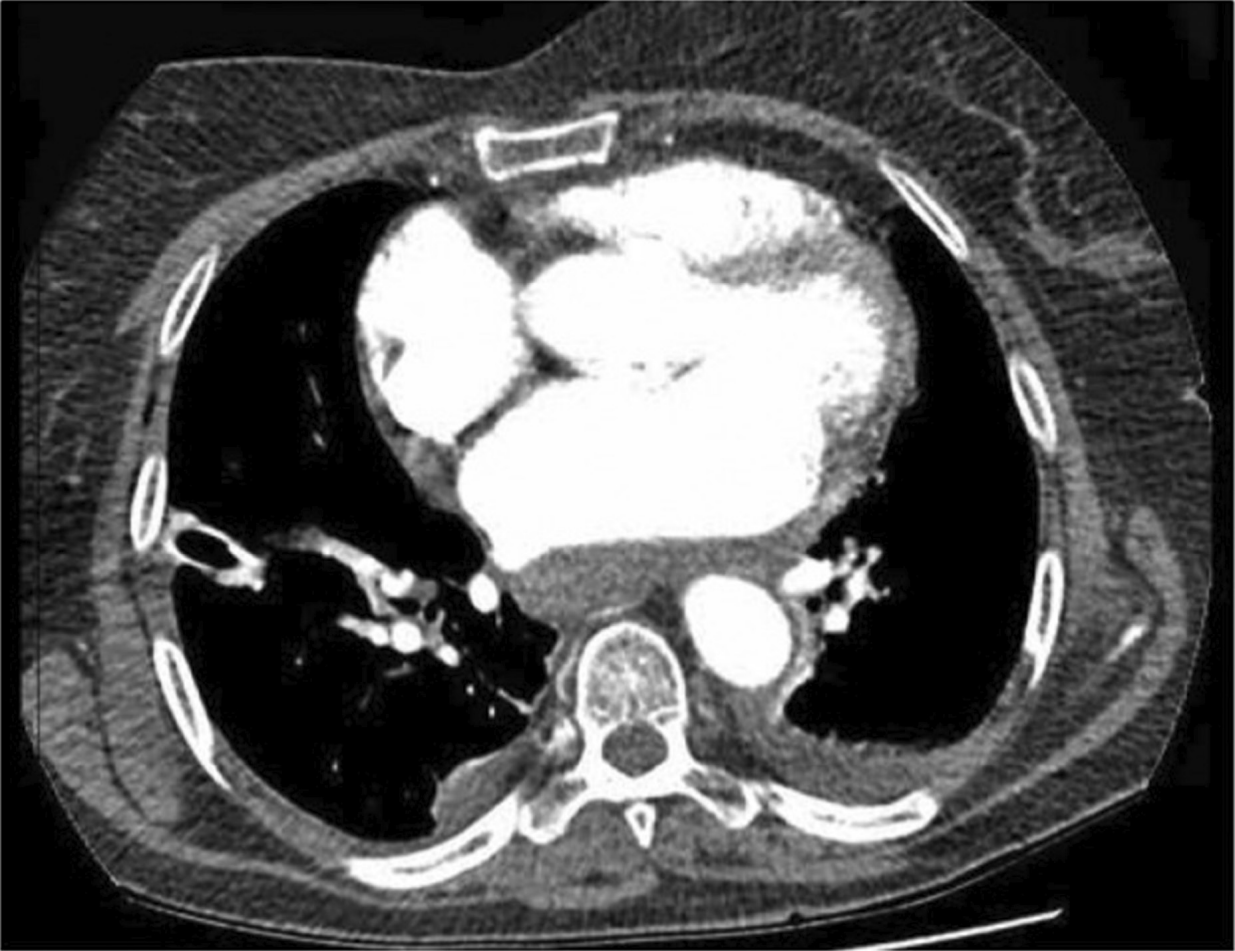
Chest CT reveals right‐side pleural effusion and mediastinal hematoma.

On the fifth day of admission, a CT angiogram showed right‐side pleural effusion and mediastinal hematoma (Figure 3). He was on a therapeutic enoxaparin dose and then switched to apixaban on the seventh day of admission, but the drain fluid continued to be serosanguineous in nature.

**FIGURE 4:**
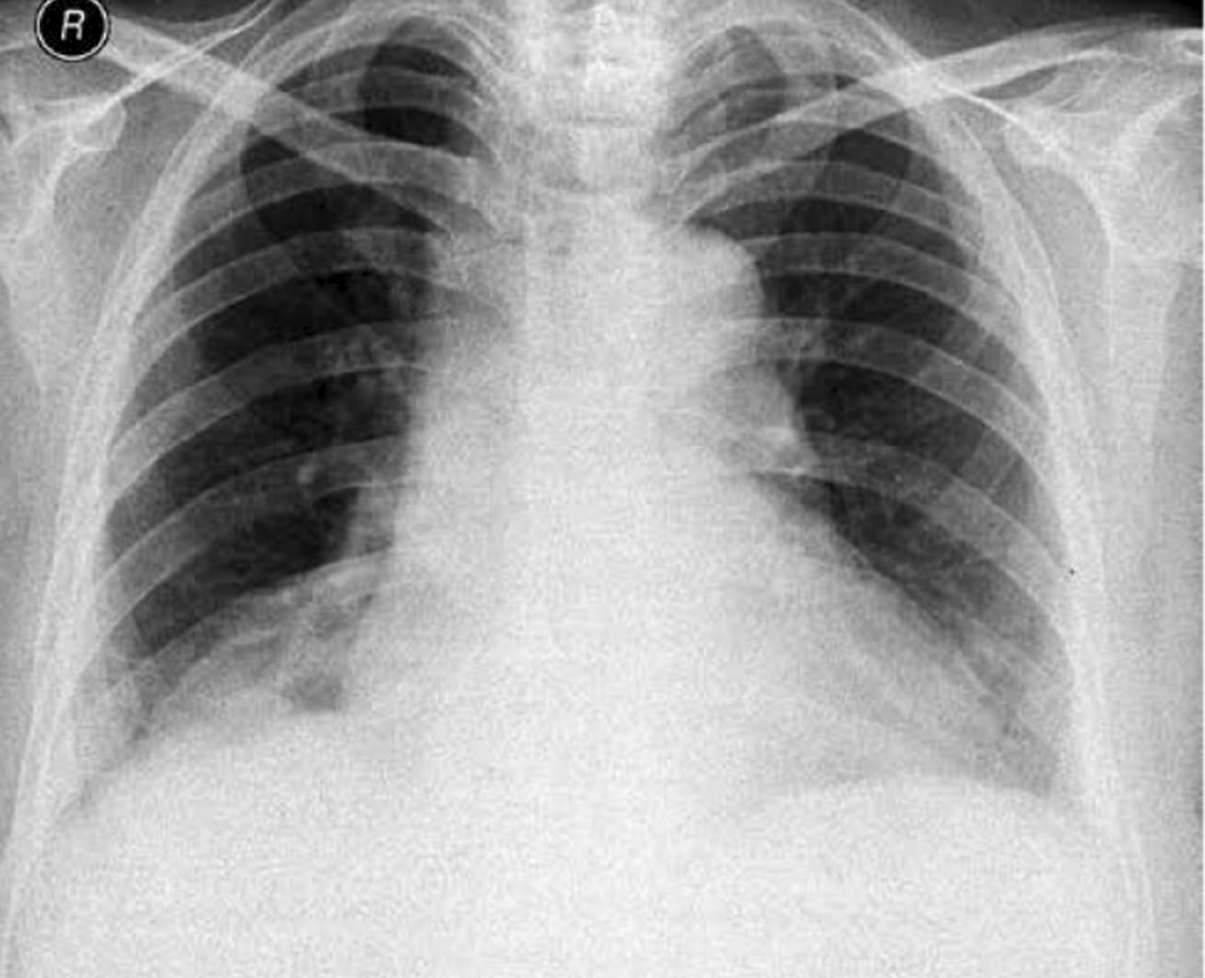
Chest X‐ray 1 month later.

On the day of discharge, the patient's chest drain was removed, and he was started on apixaban considering his high protein C level. Additionally, his anticoagulation was changed to apixaban, which he continued and monitored for any bleeding. He was also scheduled for follow‐up appointments with the pulmonology clinic. One month later, his chest X‐ray came back with normal results (Figure 4).

## 3 DISCUSSION

Patients with pulmonary infarction are more likely to experience spontaneous hemothorax related to anticoagulant medication, which typically manifests in the setting of pulmonary embolism shortly after beginning the therapy.^3^, ^6^, ^7^, ^8^ There have been reports of spontaneous hemothorax brought on by DOACs like rivaroxaban^9^ and dabigatran^5^ in the context of pulmonary embolism and atrial fibrillation, respectively.

Spontaneous hemothorax is an uncommon event. Blood building up in the pleural area without any visible injuries is known as a spontaneous hemothorax (SH). The United States Food and Drug Administration has authorized rivaroxaban as a brand‐new oral anticoagulant used for preventing recurrent venous thromboembolic disease. Factor Xa activity is inhibited by rivaroxaban. Traditional coagulation investigations are unable to determine the extent of rivaroxaban's anticoagulant action.^10^ However, rivaroxaban raises the INR when used in overdose circumstances. A linear model explains the correlation between PT and rivaroxaban plasma concentrations.^11^ There is also a dose‐dependent lengthening of the activated partial thromboplastin time.^12^ The fact that the patient's coagulation profile was within the normal range indicated that the SH was probably caused by therapeutic dosage.

The most prevalent side effects of oral anticoagulants are bleeding incidents, which can be spontaneous small artery ruptures resulting in hemothorax due to minor chest trauma, which has rarely been reported. Following anticoagulation with an outdated oral anticoagulant (warfarin), spontaneous hemothorax has been documented. Although gastrointestinal, intracranial, and soft tissue bleeding could happen.^13^, ^14^ A direct oral anticoagulant (NOAC) with a safer safety margin than warfarin is rivaroxaban. To our knowledge, no literature reports of SH related to NOAC (rivaroxaban) use exist. In this case, the development of SH in the absence of other causes suggested that rivaroxaban might have contributed to the hemothorax.

Individuals who are over 65 years old have a greater risk of bleeding events than individuals under 65 years old. Secondly, Patients with low creatinine clearance (CrCl) have a higher risk of bleeding because they are exposed to more drugs with deranged renal function.^15^ Additionally, hepatic disease patients with coagulopathy should not take rivaroxaban.^16^ For CrCl >50 mL/min, rivaroxaban is to be taken twice daily at a dose of 15 mg. Our patient was 49 years old and had neither renal failure nor a hepatic illness.

The risk of spontaneous hemothorax associated with rivaroxaban use is highlighted. Spontaneous hemothorax should be considered in patients treated with rivaroxaban if pleural fluid appears bloody or has a high hematocrit level.

## 4 CONCLUSION

After conducting thorough investigations and follow‐ups, we have identified that the primary potential cause of spontaneous hemothorax is rivaroxaban. This case is the first reported instance of concurrent spontaneous bilateral hemothorax resulting from rivaroxaban therapy. It is crucial to use NOACs cautiously and avoid their use in individuals who are at risk of bleeding. With an increasing number of patients receiving NOAC treatment, potentially life‐threatening bleeding events requiring urgent reversal are expected to rise.

## AUTHOR CONTRIBUTIONS

**Bassem Al Hariri:** Supervision; writing – original draft; writing – review and editing. **Ahmad Taher Alharafsheh:** Writing – original draft; writing – review and editing. **Muad Abdi Hassan:** Project administration; writing – original draft; writing – review and editing. **Abdulqadir J. Nashwan:** Writing – original draft. **Mohamed Gaafar Mohamedali:** Supervision; writing – review and editing. **Hatem Mabrouk Abusriwil:** Supervision; writing – review and editing.

## FUNDING INFORMATION

This case report was not funded.

## CONFLICT OF INTEREST STATEMENT

The authors have declared that no competing interests exist.

## ETHICS STATEMENT

The article describes a case report. Therefore, no additional permission from our Ethics Committee was required.

## CONSENT

Written informed consent was obtained from the patient to publish this report in accordance with the journal's patient consent policy.

## Data Availability

The data that supports the findings of this study are openly available in the references part of this manuscript.
