# Correction to “Spontaneous hemothorax caused by rivaroxaban treatment for pulmonary embolism: a case report.”

**DOI:** 10.1002/ccr3.8668

**Published:** 2024-07-11

Al Hariri B, Alharafsheh A, Hassan MA, Nashwan AJ, Mohamedali MG, Abusriwil HM. Spontaneous hemothorax caused by rivaroxaban treatment for pulmonary embolism: A case report. Clin Case Rep. 2023 Dec 11;11(12):e8333. https://doi.org/10.1002/ccr3.8333

In the article entitled “Spontaneous hemothorax caused by rivaroxaban treatment for pulmonary embolism: a case report.” that was previously published in Volume 11 Issue 12 of Clinical Case Reports, an author’s name was incorrectly formatted.

Ahmad Taher Alharafsheh’s name should be formatted as Ahmad Alharafsheh.

We apologize for this error.

